# The use of anterior subcutaneous internal fixation (INFIX) for treatment of pelvic ring injuries in major trauma patients, complications and outcomes

**DOI:** 10.1051/sicotj/2019019

**Published:** 2019-06-28

**Authors:** Richard Steer, Ganesh Balendra, Justin Matthews, Martin Wullschleger, James Reidy

**Affiliations:** 1 Gold Coast University Hospital 1 Hospital Boulevard Southport 4215 QLD Australia; 2 University of Queensland St Lucia 4072 QLD Australia; 3 Griffith University 58 Parklands Drive Southport 4215 QLD Australia

**Keywords:** INFIX, Pelvic trauma, Pelvic ring injury, Iowa Pelvic Score, Complications

## Abstract

*Purpose*: Pelvic anterior internal fixators (INFIX) are a relatively new alternative in the treatment of unstable pelvic fractures. The authors wanted to review the use of complications and outcomes of this method of pelvic fixation at our institution.

*Method:* Patients over the age of 18 who had an INFIX used in treatment of their pelvic ring injury were identified. Patient demographics, fracture type, injury severity score, morbidity, complications and time until removal were recorded. All available patients were followed up following the removal of the INFIX and completed an Iowa Pelvic Score (IPS) at this time.

*Results and Discussion*: 24 patients (19 male) with a mean age of 38.5 (range 18–71) met the inclusion criteria with an average injury severity score of 29.8 (10–66). The most common complication following insertion was a lateral femoral cutaneous nerve (LFCN) injury, which occurred in 11 patients (bilaterally in two), 6 patients (25%) had ongoing numbness 6 months post removal. Two patients had an infection, one of which prompted the removal of the INFIX. One INFIX was removed for implant failure. All other removals were planned electively. Heterotopic ossification was noted to have occurred in five cases. The mean IPS following removal of the INFIX was 79.2 (52–100). INFIX is a safe and successful treatment for unstable pelvic ring injuries. Overall, patients tolerate the INFIX well with good outcome scores. The main concern being the high rate of LFCN injuries, although many resolved after removal of the INFIX.

## Introduction

Pelvic fractures are common presentations to major trauma centres and are associated with significant morbidity in polytrauma patients. They are usually the result of high energy mechanisms such as road traffic accidents or falls from height, or the result of low energy trauma in osteoporotic bone in the elderly population. Stable fracture patterns are often treated non-operatively; however, unstable patterns require surgical fixation due to the significant morbidity and mortality associated with pelvic fractures and their associated injuries [1–3].

Traditional open reduction and internal fixation is associated with a high incidence of surgical morbidity, while external fixators, used for both temporary stabilisation and as definitive management, have a complication rate of up to 62% [4], with poor patient tolerance, pin site infection and aseptic loosening the more commonly documented complications in the literature [4, 5]. Recent literature suggests that pin site infections occur in 18% of pelvic injuries treated with external fixation, with much higher numbers reported in older literature. This has resulted in the use of pelvis external fixation to be limited to emergency trauma settings or when open reduction internal fixation is precluded [6].

Minimally invasive techniques have become more popular recently in the management of pelvic injuries due to their lower incidence of surgical morbidity. The application of a pelvic internal fixator (INFIX) has been presented as a comparable alternative to external fixation of anterior pelvic ring injuries [7, 8]. An INFIX involves the insertion of spinal pedicle screws in the anterior pelvis (supra-acetabular entry) and the placement of a connecting rod in the subcutaneous tissue of the patient [5]. The INFIX may be augmented with other pelvic fixation as dictated by the nature of the injury. The INFIX is generally removed at a minimum of 3 months after insertion, once fracture union has occurred.

The earliest description of an INFIX was published in the German literature in 2009 [9] with a subsequent mid-term follow-up cohort from the same authors in the English literature in 2013 [8]. Scheyerer et al. [10] describe the INFIX technique in a technical note as well as the theoretical advantages for this technique over external fixation, which include reduced infection risk, patient mobility and nursing requirements.

Indications for use of INFIX are not well defined, but most authors reserve the use of the technique for unstable pelvic injuries (Young and Burgess Classification: Vertical Shear, APC 2, APC 3, LC2, LC3). Complications associated with the use of INFIX are well described within the literature. A recent systematic review by Vaidya et al. [11] of 496 patients treated with INFIX found reported complications including lateral femoral cutaneous nerve (LFCN) injury/irritation (26.3%), heterotopic ossification (36%), infection and wound complications (3%) and femoral nerve palsy (1%). Device loosening and patient comfort concerns have also been reported by other authors.

There is minimal current literature on patient subjective follow-up with questionnaires about daily activity and function following the application (and subsequent removal) of the INFIX. The systematic review by Vaidya et al. [11] found only six articles reporting outcome score for a combined 197 patients. One used a German pelvic outcome score while the others used the Majeed Pelvic score, with 87 excellent, 77 good and 33 fair results. A systematic review of pelvic fracture scores by Lumsdaine et al. [12] compared the Iowa Pelvic Score (IPS), Majeed Pelvic Score and the Orlando Pelvic Score against more recognised outcome scores like SF-36. All of these outcome scoring systems were effective and easy to administer. The IPS appears to have the best correlation to the SF-36 and is a good tool for measuring outcomes in severe pelvic injury [13].

This study retrospectively reviews the outcomes of patients with unstable pelvic injuries (excluding isolated symphyseal disruptions) managed with an INFIX at a major trauma centre in Australia.

## Methods

Approval of this study was obtained from our institutions Human Research Ethics committee. The researchers retrospectively and prospectively collected data and outcome scores on all patients who had a pelvic INFIX used for treatment of their pelvic ring injury at our institution, a level-one trauma centre, from 2014 to 2017. The start date of the surgical technique correlated with the employment of a new pelvic trauma surgeon (JR) who had learned the surgical technique at another institution. All operations were performed under the supervision of the senior author in a method similar to that described by Vaidya et al. (2012).

Our institution used two different brands of INFIX devices: eight Malibu (Seaspine, Carlsbad, CA, USA) and 16 Nuvasive (San Diego, CA, USA). Both systems use a poly-axial head. While a polyaxial head decreases the overall strength of the construct, it allows the joining rod to be placed in the optimal position to limit abdominal impingement.

Inclusion criteria were patients over the age of 18 with unstable pelvic injuries treated with an INFIX. Patients were identified via searching a combination of the hospital’s operating room management system (ORMIS) and the local trauma service registry. Data were then gathered from patient electronic records, radiology and ORMIS records. With ethics approval, all available patients were contacted to gather further information on complications of the INFIX, general tolerance of the device and to document an IPS.

Lower limb neurological assessments were undertaken as part of usual assessment on all patients before and after their surgery. This helped delineate any neurological changes related to the injury or the device implanted. All patients had a medical history taken, including previous abdominal or groin surgery and presence of known hernia. While there were no patients in our cohort that had a hernia or previous surgery on their lower abdomen, this would be a relative contraindication to the use of the INFIX.

The main outcome measure for the study was successful fracture union, with secondary measures include the IPS and complications from the use of an INFIX. Data were also collated on timing of surgery, thromboembolic disease and inferior vena cava (IVC) filters, injury severity scores (ISS), length of intensive care unit (ICU) stay, mechanism of injury, classification of pelvis injury and other fixation required for pelvic stability. Data were collected on all patients who received an INFIX with a minimum 6 months post initial surgery. Outcome scores were obtained on all participants who consented to further data collection at a minimum 3 months post removal of INFIX and followed up clinically for at least 12 months. General tolerance of the device including abdominal discomfort when sitting or leaning forward, while in place was also gathered from outpatient notes and on questioning at follow-up or while completing the IPS.

## Results

Twenty-four patients (19 male, 5 female) met the inclusion criteria with a mean age of 38.5. The most common cause of injury was motor vehicle accidents evenly divided between cars (29%, *n* = 7) and motorbikes (29%, *n* = 7) followed by a fall from height (25%, *n* = 6). The mean ISS was 29.8 (range 10–59). The classification of the pelvic injuries in our cohort can be seen in Table 1. The operations were performed by surgeons of varying levels of experience including an orthopaedic pelvic trauma surgeon, a general trauma surgeon, a general orthopaedic surgeon and senior orthopaedic registrars (trainees) under supervision of the pelvic trauma surgeon.

**Table 1:** Pelvis injury pattern – young and burgess classification.

Fracture type	*n* (%)
Lateral compression 2	7 (29)
Lateral compression 3	3 (13)
Vertical shear	7 (29)
Anterior-posterior compression 2	2 (8)
Combined mechanism	5 (21)

The cohort included 10 (42%) unstable lateral compression fractures (LC2 and LC3 injuries) making up the majority of cases, seven (29%) vertical shear type fractures, two APC2 pelvic injuries, and five (21%) combined fracture types that were complex unstable injuries, often a combination of lateral compression and vertical shear that did not fit the Young and Burgess classification. All patients had a bony injury to the anterior pelvic ring (pubic rami or body). Isolated pubic symphysis injuries were excluded from this study as they were treated either conservatively or with symphysis plating.

**Figure 1:**
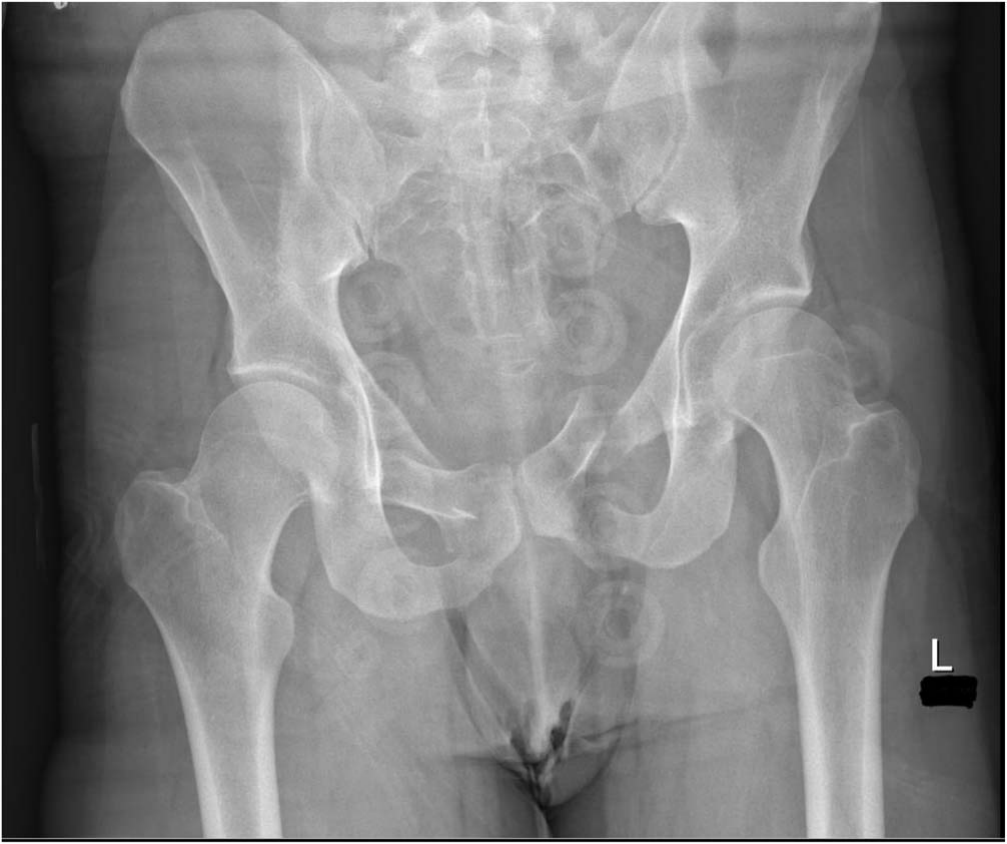
Initial imaging of a combined mechanism pelvis injury.

**Figure 2:**
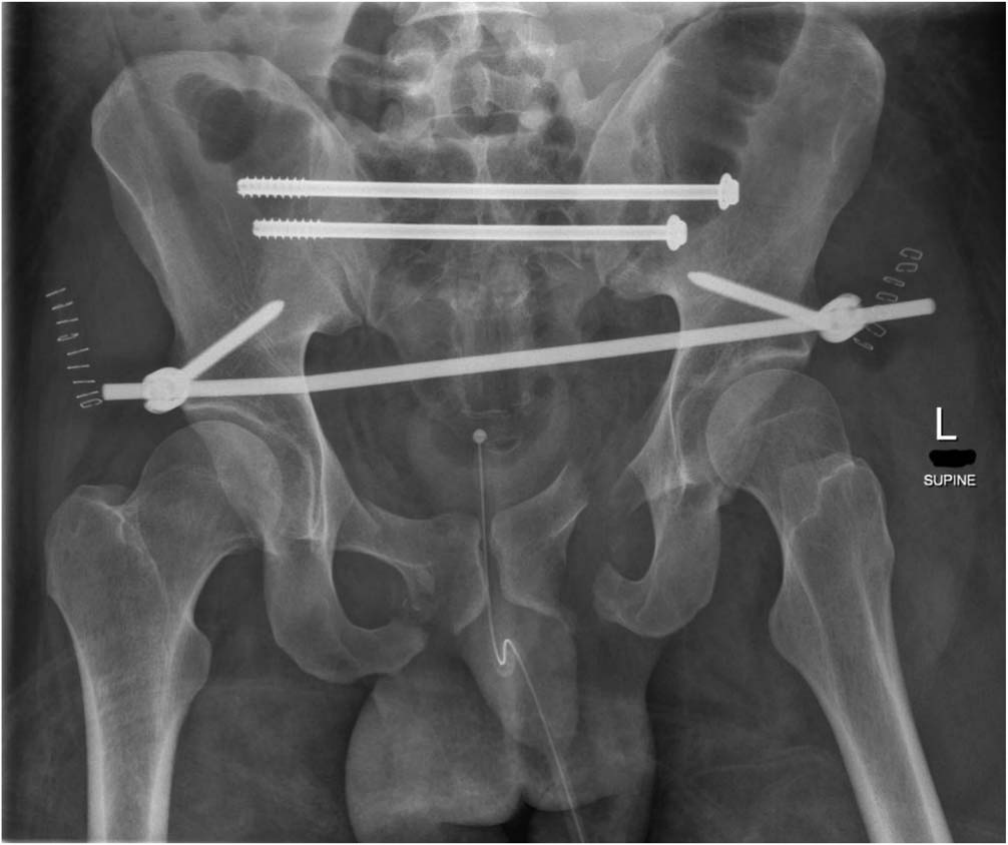
Day 1 post pelvis fixation: trans-sacral S1 and S2 screws with INFIX.

**Figure 3:**
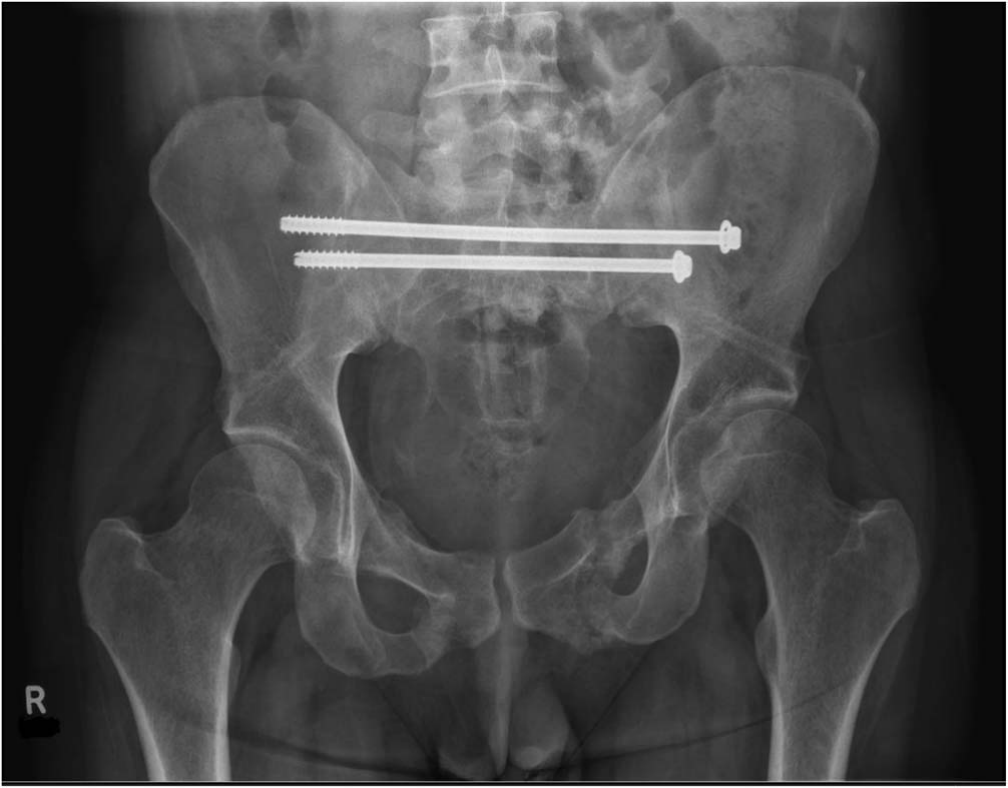
After removal of INFIX and anterior union.

Figures 1–3 – arrival, post-op and post-removal images of the use of an INFIX.

Sixteen patients were direct presentations to our institution and eight patients were transferred from regional centres. All operations were performed for acute injuries with a mean time to theatre of 5.2 days (0–13). Patient’s required a mean intensive care unit (ICU) stay of 6.8 days (0–49) and mean length of total hospital stay of 28.8 days (8–118).

**Figure 4:**
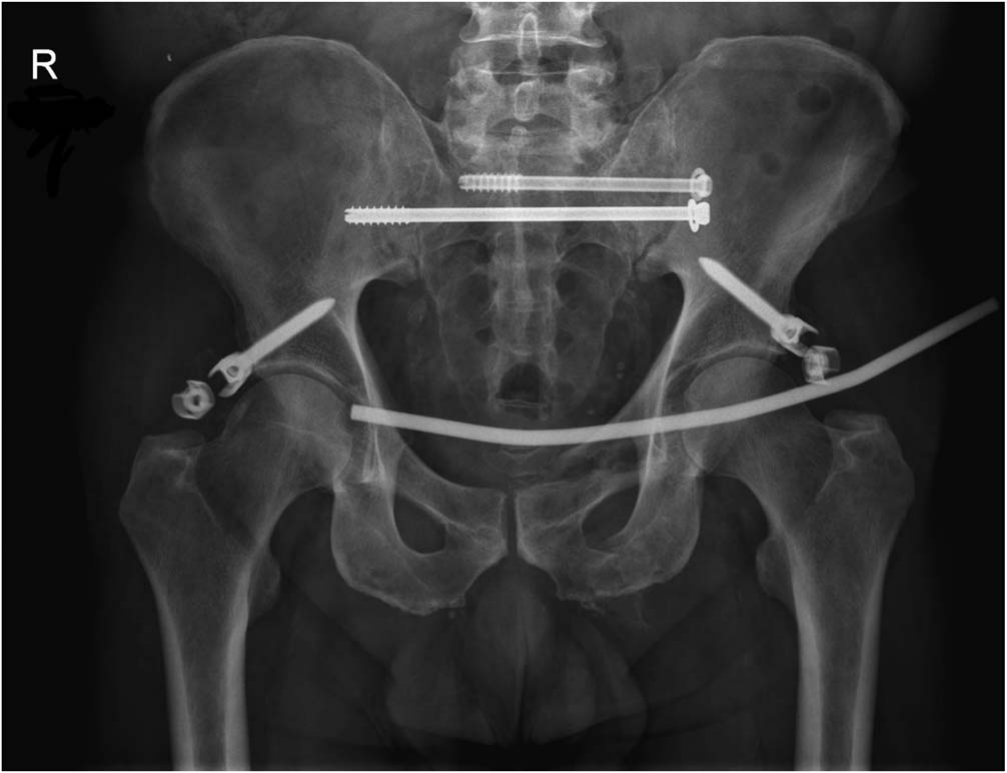
Metalware failure: device locking mechanism failure.

The mean time to removal was 20.4 weeks. One patient was lost to follow-up with an INFIX in situ, as they were repatriated overseas with their INFIX in situ. One patient was lost to follow-up following removal. One INFIX was removed for infection and one for implant failure (Figure 4). The implant failure was as a result of the locking screw coming loose on the device. This failure prompted the change of implant used, no further failures have occurred since. The pelvis was healed at the time this occurred, so the INFIX was removed with no further treatment required. All other INFIXs maintained adequate fracture reduction, with all anterior pelvic ring injuries united in a satisfactory position. The remaining INFIXs underwent planned removal after union. Removal of the implant takes approximately 30 min, is a day case, patients were allowed to continue full weight bearing afterwards and there were no known complications associated with removal.

Fifteen patients (63%) were contactable and consenting to the completion of an IPS at least 3 months following removal. The mean IPS was 79.7 (52–100), five excellent (85–100), seven good (70–84), two fair (55–69) and one poor (<55).

The most common complication of INFIX insertion was LFCN injury which occurred in 11 patients, two bilateral. This was 13 LFCNs affected out of 48 (27%) or 46% of patients initially. After removal, many of these resolved with 7/48 (14.6%), six patients total (25%), persisting at their most recent follow-up. Five patients had evidence of heterotopic ossification (21%) and two patients had wound issues, being wound dehiscence and superficial infection. Four patients (17%) had a venous thromboembolism (VTE) during their initial inpatient stay. In our study, there were no incidents of femoral nerve injury. Overall, the INFIX was well tolerated with just four patients complaining of discomfort from the implant, usually with sitting or leaning forward causing lower abdominal discomfort.

## Discussion

Figures 5–12: Series of images of case example, from initial arrival in pelvic binder to final union after removal of INFIX.

**Figure 5:**
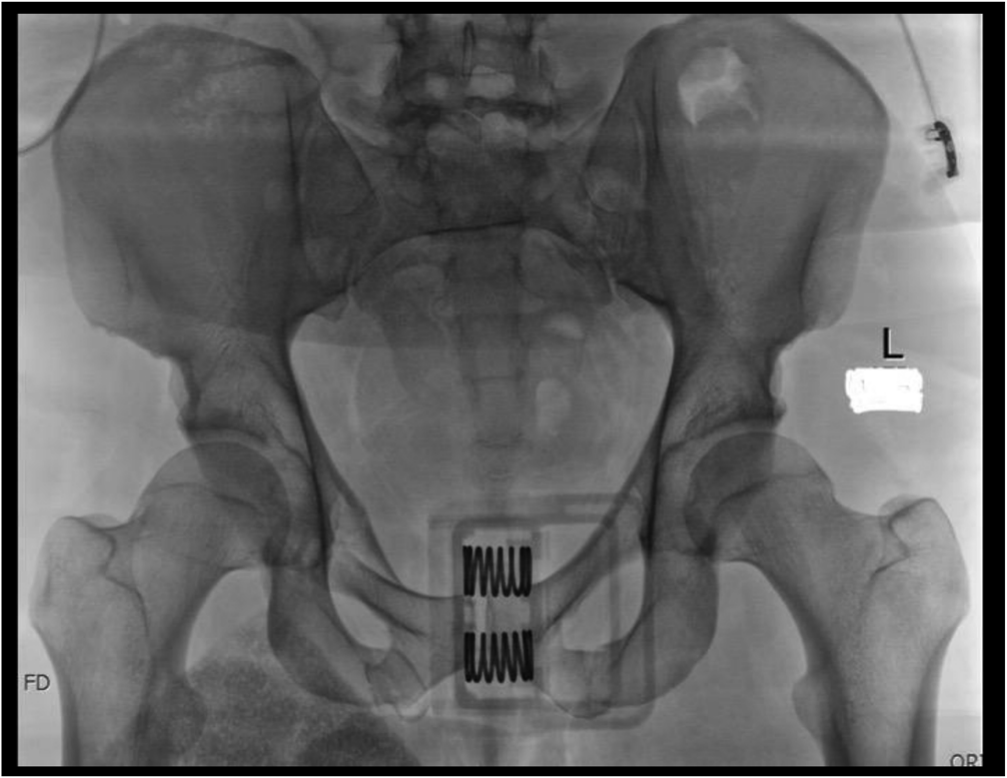
Straddle type fracture-bilateral public rami fractures with bilateral sacral fractures. Initial radiograph on arrival with binder on.

**Figure 6:**
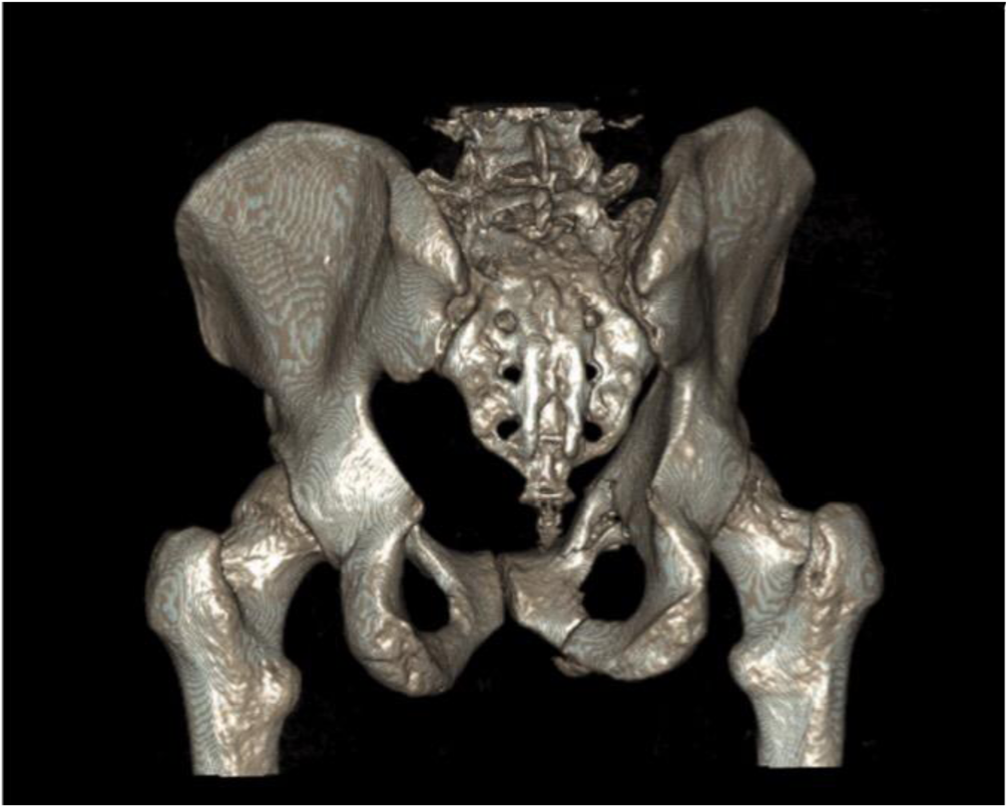
CT 3D reconstruction of Figure 5 injury.

**Figure 7:**
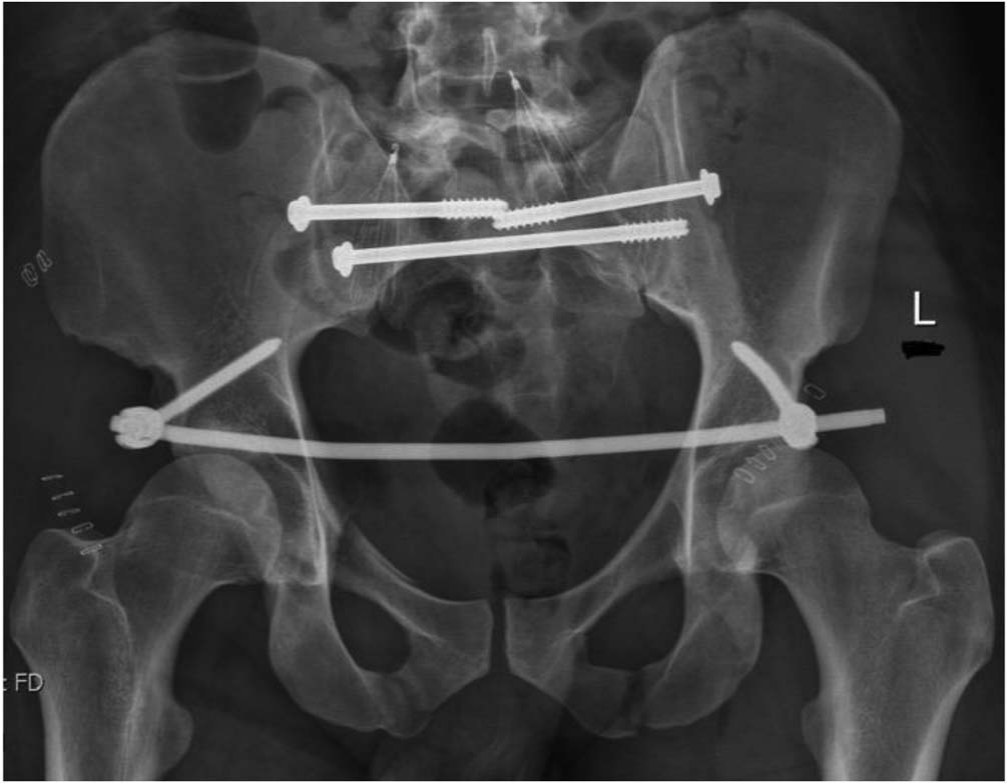
Day 1 after fixation. Bilateral sacroiliac joint screws with INFIX. Filters in both common iliac veins. AP image.

**Figure 8:**
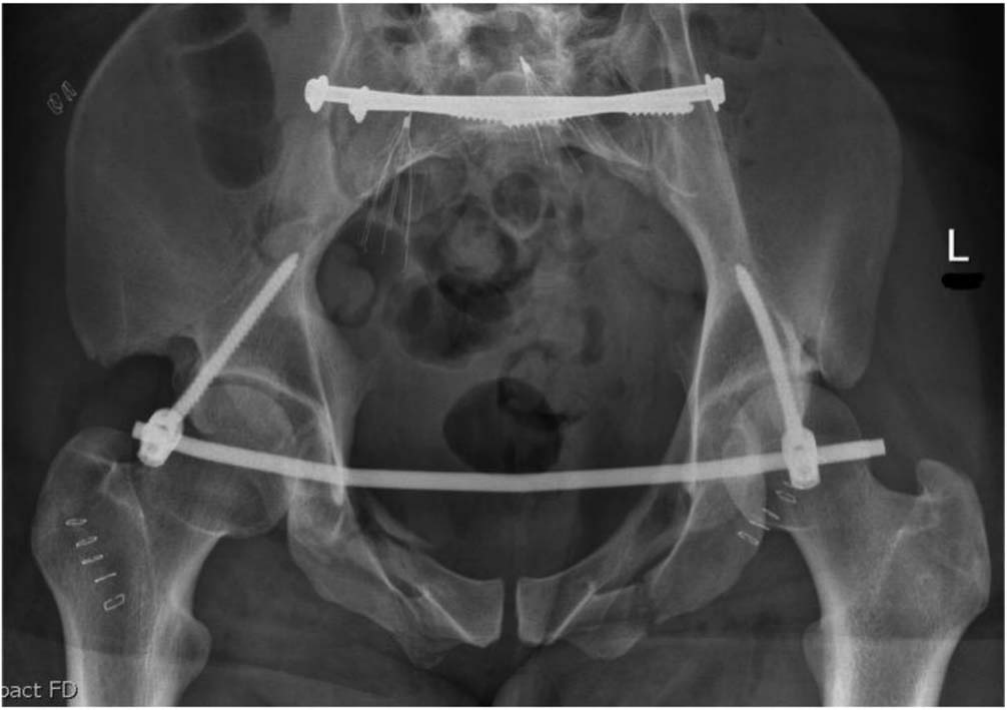
Day 1 after fixation. Bilateral sacroiliac joint screws with INFIX. Inlet image.

**Figure 9:**
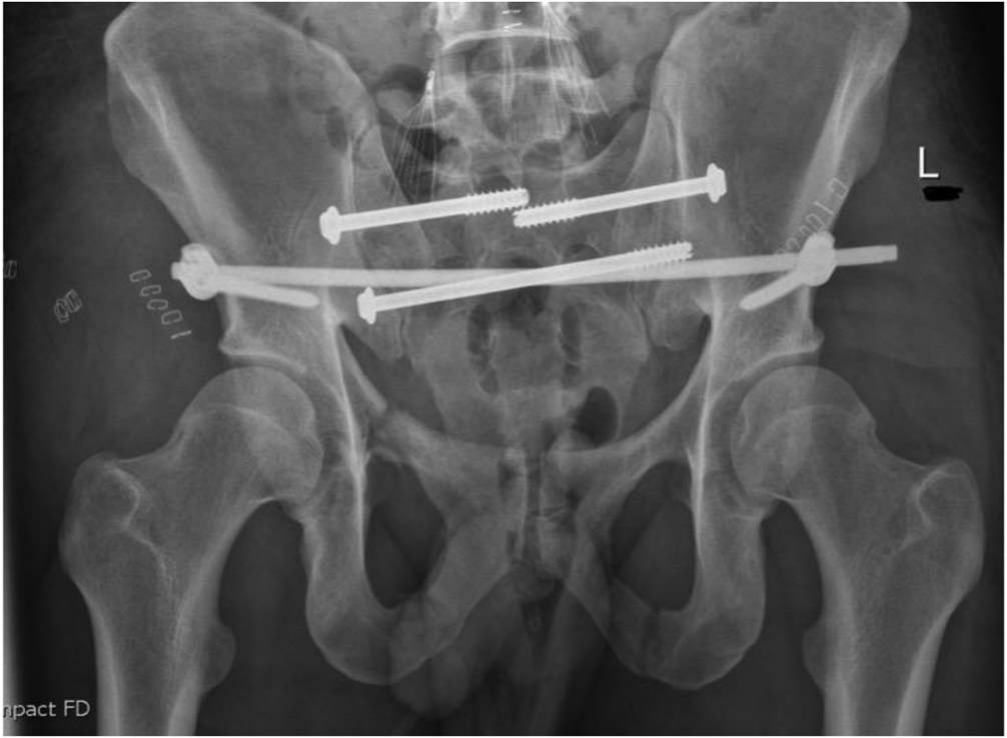
Day 1 after fixation. Bilateral sacroiliac joint screws with INFIX. Outlet view.

**Figure 10:**
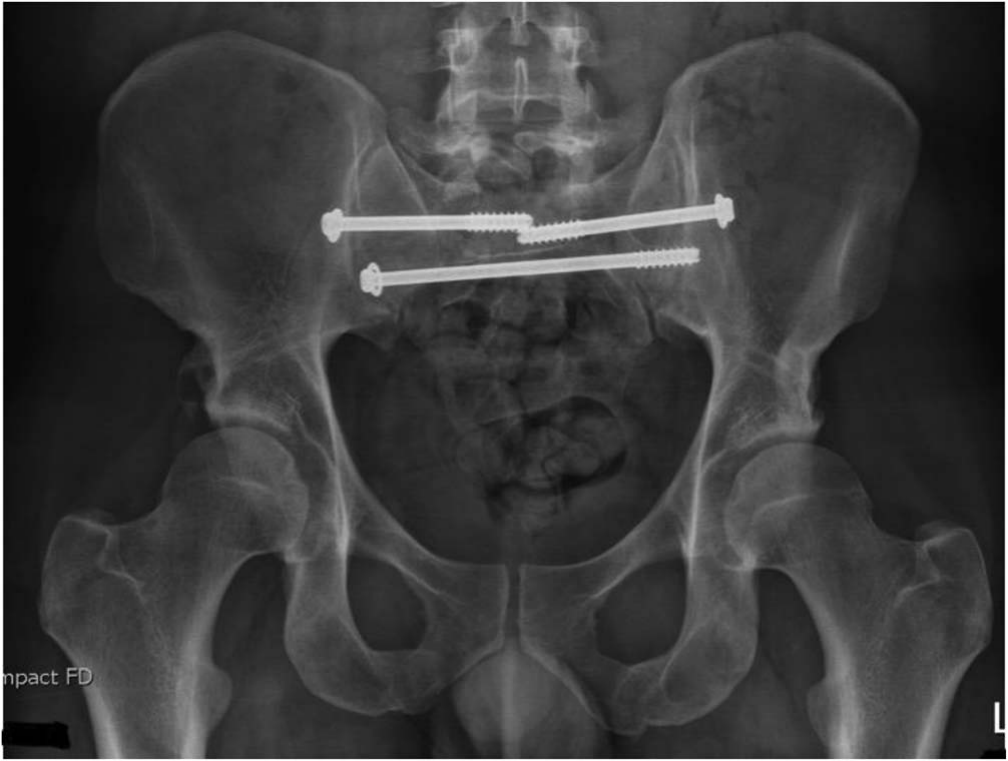
After union and removal of INFIX. Mild right-sided heterotopic ossification noted. AP image.

**Figure 11:**
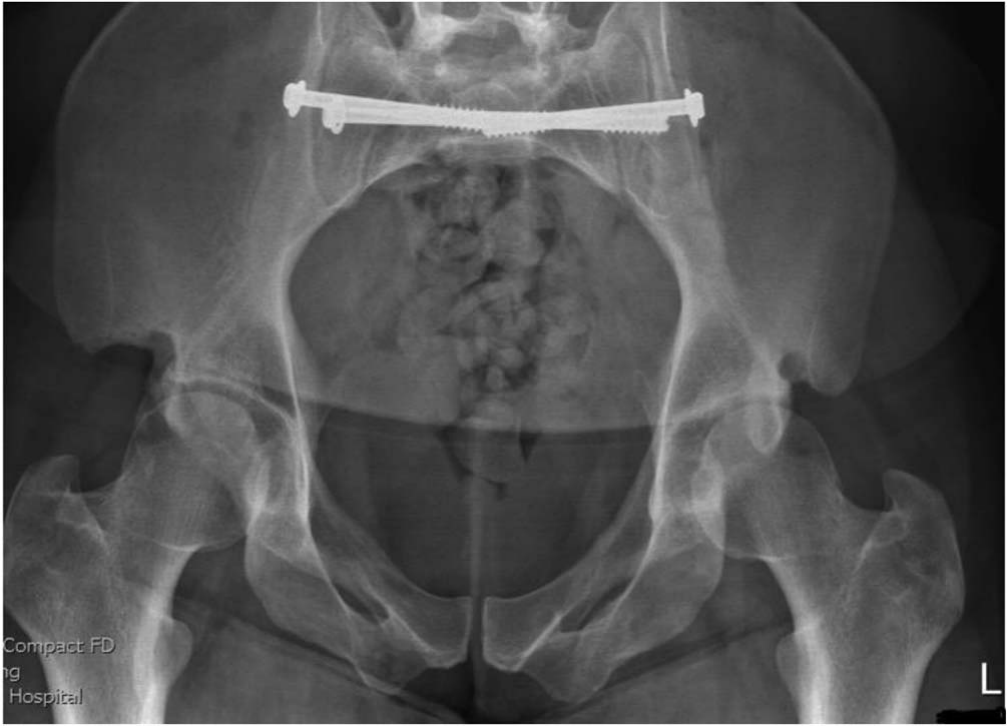
After union and removal of INFIX. Mild right-sided heterotopic ossification noted. Inlet image.

**Figure 12:**
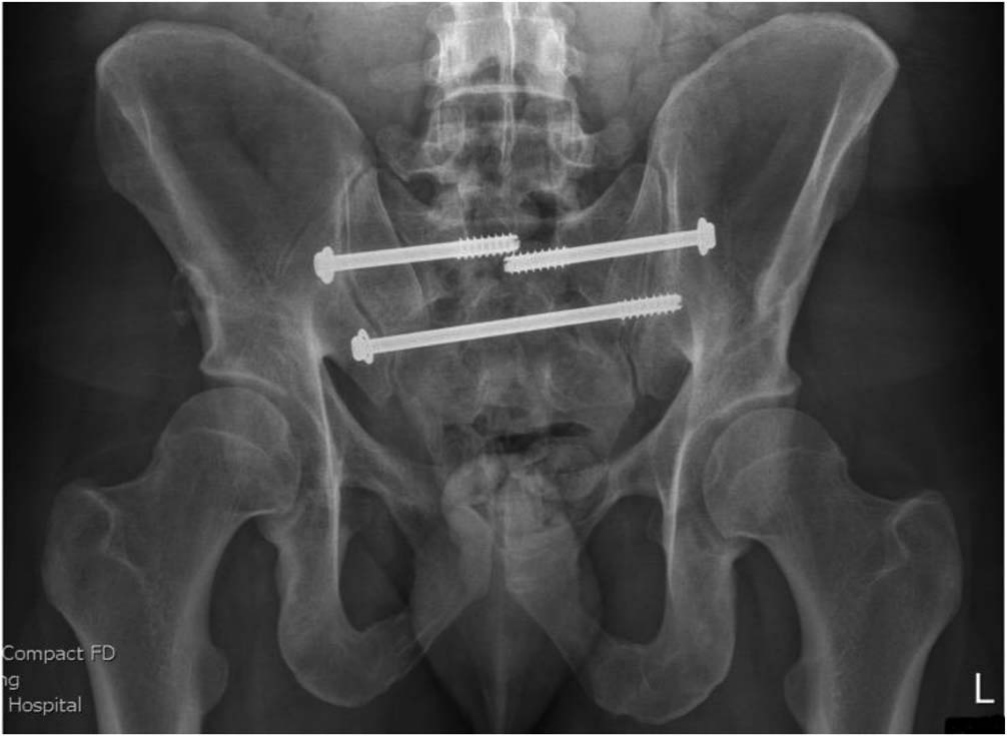
After union and removal of INFIX. Mild right-sided heterotopic ossification noted. Outlet image.

This study adds to the body of literature currently available on the use of an INFIX in the management of unstable anterior pelvic ring trauma. The INFIX is an easily reproducible technique, and was performed in our series by a total of eight surgeons (three consultants and five senior registrars) under the supervision of the senior author. The heterogenous nature of those performing the operations confirms the suitability of using this surgical technique in a broader orthopaedic community. In our experience, the presence of an experienced radiographer as part of the surgical team is invaluable in obtaining appropriate intraoperative images, and makes the operation easier.

Müller et al. [8] published the first series on the results in INFIX in 36 patients who had a Type C pelvic fractures (excluding symphysis rupture) on the Tile classification. They measured clinical and radiological results over a 7 year period, though each patient was only followed up for 18 months. 31 patients (86%) were followed up to completion of the study. Three patients passed away within the perioperative period due to multi-organ failure. The INFIX was removed at an average of 9 months post insertion. Thirty patients had union of fracture at time of removal. They noted that at final follow-up, all patients have significantly worse SF-12 scores compared to the general population.

Vaidya et al. [14, 15] published initial and mid-term results of the use of INFIX in pelvic injuries. In total they had 83 patients over a 7-year period with average 3-year follow-up. They included Lateral Compression Type one (LC1) pelvic fractures, often considered a stable fracture pattern with a general consensus for non operative management, as well as isolated symphyseal injuries, which we maintain should be treated with open reduction and internal fixation. They had a loss to follow-up of 25% of their cohort, and a further four patients who passed away. Vaidya et al. [14] also looked at results from a multicentre study of 91 patients at four level-one trauma centres over a 3-year period. Six patients had early revision surgery, which may be related to unfamiliarity to the device itself.

Wang et al. [16] compared INFIX to open reduction and internal fixation in Type B (Tile classification) pelvic fractures and found the INFIX superior in operation time, length of hospital stay as well as in clinical and radiological 6-month follow-up. However, no subjective questionnaires were given to the patient.

Fang et al. [17] retrospectively reviewed 43 patients at their level-one trauma centre between 2012 and 2015. Seventeen were lost to follow-up; however, three had complications within 3 months and were included in their cohort leaving 29. Most complications were in keeping with other studies, however of note they had one femoral nerve palsy. This is a complication only seen in one other series by Hesse et al. [18] which had a femoral nerve palsy in six of their eight cases. All patients underwent femoral vessel duplex ultrasound confirming patency of the femoral vessels post-operatively, this was not part of the routine check on our patients. Fang et al. did not routinely remove the INFIX from patients and 27.5% of their patients elected to keep the implant in to avoid another operation.

Complications of the INFIX are well described. The more frequent complications noted are Heterotopic Ossification (HO), LFCN injury, Infection and thromboembolic events. In a cadaver study by Reichel et al. [19] comparing the pelvic bridge and INFIX to local anatomic structures, they found the LFCN to be an average of only 2.2 mm away from the INFIX screw. This would certainly explain the high rate of LFCN injury noted by all authors. Our technique, in keeping with Vaidya et al. [20] involved blunt dissection between the tensor fascia lata and sartorius muscles with careful attention to retract the LFCN. Despite protecting the nerve, its close proximity to the device location means it likely ends up travelling around the screw or compressed by the device with sitting, causing the neurological symptoms. All authors have encountered this problem with no clear way to avoid it occurring, rather aim to minimise trauma to the LFCN.

In our studies, rate of LFCN injury/irritation substantially dropped from the initial 11 patients to only six after removal (25%). This number is in keeping with other studies, including the systematic review by Vaidya et al. [11] with an overall rate of 26.3% for all published studies. The exact rate in other published studies can sometimes be difficult to interpret as there is variance in reporting between number of patients versus the rate compared to the number of LFCNs. Thus we have included both for clarity. None of our patient cohort had ongoing neuralgia after removal, rather just ongoing numbness. With further time, we would expect more of the patients would have resolution to their numbness, minimum 6 months follow-up being too soon to know the final potential improvement.

Heterotopic ossification (Figure 10) occurred in 21% of our patients. It was noted on follow-up radiographs immediately around the screw site at the AIIS. With a minimum 12-month follow-up, no patients had ongoing symptoms or had required further treatment of the heterotropic ossification. We did not routinely use any heterotopic bone prophylaxis, i.e., NSAIDs. This is something that authors could consider studying in future.

Pelvis injuries and severe trauma patients are known to have high rates of VTE, up to 61% [21]. At our institution, all patients are started within 12 h on low molecular weight heparin or unfractionated heparin unless there is contraindication to use, i.e., active bleeding or intracranial haemorrhage. These patients then had an inferior vena cava filter placed for prophylaxis as soon as stable enough for the procedure. All patients had regular lower limb screening ultrasound scans to monitor for deep vein thrombosis (DVT). VTE prophylaxis was continued for 12 weeks as per guidelines by El-Daly et al. [21]. Our cohort had a rate of 17% for VTE, in a severe trauma population, we consider this rate to be on the lower end. In our routine VTE, all patients with thromboembolism underwent a period of 3–6 month treatment with anticoagulation at the discretion of our haematologists. Inferior vena cava filters were removed routinely once patients were mobile, able to have usual chemical prophylaxis and deemed to be at lower risk.

This study has a number of limitations that we acknowledge. Ours is a retrospective study with small number of patients. This study suggests that by proving that INFIX application is a reproducible technique with a relatively small complication in comparison to traditional external fixation, we can expand the technique to wider range of pelvic injuries, as other authors have done.

The pelvic INFIX is a safe and reproducible technique for management of unstable anterior pelvic ring injuries. This study suggests that this treatment technique is a successful treatment option in more severe pelvic trauma with similar complication rates to previous studies. The most significant noted complication is that of LFCN nerve injury, this is in keeping with all other authors having published on INFIX. The outcome scores are better than those shown in previous studies looking at traditional fixation methods. The need for a prospective multicentre study with comparison against other fixation methods is still warranted to validate this form of fixation as a better alternative to traditional methods.
